# Childhood cancer incidence in relation to sunlight exposure

**DOI:** 10.1038/sj.bjc.6606015

**Published:** 2010-11-23

**Authors:** J R B Musselman, L G Spector

**Affiliations:** 1 Division of Pediatrics, Department of Epidemiology and Clinical Research, University of Minnesota, Minneapolis, MN 55455, USA; 2 Masonic Cancer Center, University of Minnesota, Minneapolis, MN 55455, USA

**Keywords:** vitamin D, sunlight, childhood cancer

## Abstract

**Background:**

There is increasing interest in the possible association between cancer incidence and vitamin D through its role as a regulator of cell growth and differentiation. Epidemiological studies in adults and one paediatric study suggest an inverse association between sunlight exposure and cancer incidence.

**Methods:**

We carried out an ecological study using childhood cancer registry data and two population-level surrogates of sunlight exposure, (1) latitude of the registry city or population centroid of the registry nation and (2) annual solar radiation. All models were adjusted for nation-level socioeconomic status using socioeconomic indicators.

**Results:**

Latitude and radiation were significantly associated with cancer incidence, and the direction of association was consistent between the surrogates. Findings were not consistent across tumour types.

**Conclusion:**

Our ecological study offers some evidence to support an association between sunlight exposure and risk of childhood cancer.

Increasing interest has been given to the health effects of ultraviolet B exposure and its association with subsequent production of vitamin D, including the association between vitamin D and cancer incidence and mortality. Vitamin D regulates many genes that control cell growth, inhibit angiogenesis, and control cell differentiation (Egan, 2009; Garland *et al*, 2009). Epidemiological evidence suggests an inverse association with vitamin D status and/or sunlight exposure and adult cancers in several sites (Jemal *et al*, 2000; Freedman *et al*, 2002; Boscoe and Schymura, 2006; Lappe *et al*, 2007; Grant and Mohr, 2009) as well as with retinoblastoma in children (Jemal *et al*, 2000). Sunlight exposure is the primary source of vitamin D for humans (Adams *et al*, 1982; Garland, 2009) and levels are known to vary with sunlight exposure (Egan *et al*, 2005, 2008; Porojnicu *et al*, 2007). Evidence suggests that overall sunlight exposure – and therefore vitamin D status – varies in predictable ways at the population level (Garland *et al*, 2009; Grant and Mohr, 2009) and in particular with a link between residential latitude, sunlight exposure, and vitamin D status (Garland *et al*, 2006; Grant and Mohr, 2009).

None of the studies have investigated a possible association between sunlight exposure and cancer incidence in children. We conducted an ecological study of this question using registry data from the IARC publication International Incidence of Childhood Cancer, Vol. II (Parkin *et al*, 1998).

## Materials and methods

Data on incident cancer rates were extracted from the International Incidence of Childhood Cancer, Vol. II, which represents a compilation of data from 75 childhood cancer registries in 57 countries ranging in registry coverage area from single cities to entire nations. Some countries such as the United States contributed multiple registries including a race-specific registry in the Los Angeles area, and data from the multi-state SEER program. For each registry, we extracted person-years and counts of incident cases stratified by sex and age. Categories for age stratification were age <1, 1–4, 5–9, and 10–14 years at diagnosis. As many registries did not separate ages <1 and 1–4, we collapsed these strata for comparability across registries; thus for each registry we recorded a total of six counts and six person-years – one for each of the gender/age strata.

As direct measurements of individual sunlight exposure were not possible, we employed two population-level surrogate measures that are associated with sunlight. The first is the absolute latitude – distance north or south of the equator; and we used either the latitude of the city listed as the site of the registry, or the registry's population centroid if no site was listed. If a city was listed, we took its geographical central latitude and assumed that there would not be sufficient variation in latitude within a city to justify using its population centroid. The population centroid is defined as the geographic centre of the population mass for a given area and can thus be thought of as the ‘average’ geographical location of that population. Centroids for each registry were calculated as average latitude and longitude weighted by population using data from the Socioeconomic Data and Applications Center (Center for International Earth Science Information Network, 2005). Latitudes were categorised into bands of increasing distance from the equator, each band having a width of 10° the band closest to the equator (0–10°) served as the reference.

Our second surrogate marker was global solar radiation, the total amount of solar radiation that reaches the Earth's surface which is a direct measure of the sunlight actually reaching a particular point on the Earth (Glickman, 2000). Average global radiation was collected from the Atmospheric Data Center at NASA as aggregate measures based on measurements over the 22-year period from 1983–2005 (NASA, 2005). Average global radiation was extracted for each month and then summed over all 12 months to get an estimate of relative annual global solar radiation. This measure was then categorised into annual solar radiation that was low (0–45 kWh m^−2^), moderate (>45–60 kWh m^−2^), and high (>60 kWh m^−2^).

Economic disparities between registry host nations can influence the quality and completeness of registry data. In our international comparisons, we collected data on several indicators of social and economic health and equality for each registry nation, namely gross domestic product (GDP), the GINI index (a measure of equality in the distribution of a nation's wealth, with a lower index indicating more equal distribution), male and female literacy rates, infant mortality rates, and male and female life expectancy (UNESCO Custom Tables, UNESCO Institute for Statistics. World Development Indicators and Global Development Finance, World Bank Group, 1952, 1959, 1963, 1967, 1970, 1973, 1977, 1982, 1983, 1985, 1987, 1990, 1996).

The early age of onset of childhood cancers, among other lines of evidence, suggests that initiation of these cancers may occur *in utero* as well as postnatally (Ross *et al*, 1996; Paltiel *et al*, 2004; Chen *et al*, 2005; Ross, 2008). We therefore defined the time of exposure as beginning at conception and lasting until the age at diagnosis, as this time frame encompasses the entire span in which sunlight exposure could affect cancer risk. This time frame is thus dependent upon the child's age of diagnosis and the years that the registry ascertained cases. We were not able to be entirely precise in our exposure window estimates because the available data contained neither information on length of gestation nor the exact age at diagnosis – only an age stratum. For example, any case in the 5–9 age stratum could have been 5, 6, 7, 8, or 9 years old at diagnosis and thus could have been *in utero* approximately 6, 7, 8, 9, or 10 years ago. Furthermore, a child in the 5–9 age stratum could have been diagnosed in the first year, the last year or any intermediate year of the registry's existence. If we determined the time that the oldest (the child at the maximum age in the stratum diagnosed in the first year of the registry) case would have been *in utero*, the years spanning from that time point until the closing year of the registry would cover the exposure window of all of the cases in that age stratum, and that time frame would then be the exposure window as we defined it. We performed these calculations for each registry for each of the three age strata (0–4 years, 5–9 years, and 10–14 years).

Once we established each stratum/registry specific window, we recorded each socioeconomic variable for every available year of that window. We then found an average (mean) for each variable, and then, within each stratum, categorised each socioeconomic variable into quartiles. Thus, for each socioeconomic variable, each registry had three age-specific quartile rankings (0–4 years, 5–9 years, and 10–14 years). Correlations among these measures were assessed to address multicollinearity issues that would arise from adding overly correlated measures into the same model. A set of relatively uncorrelated economic indicators would be selected *a priori* to be entered into the analysis model to adjust for economic and social inequality between and within registry nations.

### Statistical analysis

As over-dispersion of the extracted count data would make the use of the Poisson distribution inappropriate, negative binomial models were fitted first, as this distribution estimates a dispersion parameter. Log-likelihood tests were performed to assess whether this dispersion parameter was non-zero, and if not the Poisson model was fit. If evidence of over-dispersion was found (i.e., a dispersion parameter not equal to zero), the negative binomial model was retained. An offset of the natural log of the person-years was used to account for the case denominator. Person-years were not available for three registries (Pakistan, the United Arab Emirates, and Papua New Guinea), and were dropped from our analyses. Case counts were separate for each gender/age stratum as totals for the duration of the registry, so for each registry a total of six counts (two gender by three age strata) and person-years were used in each model. All models were fit using SAS software (Cary, NC, USA).

As annual solar radiation and latitude were negatively correlated (*r*=−0.74, *P*<0.0001), separate models were fit for each sunlight indicator to avoid multicollinearity. Models were fitted for each of the 12 primary ICCC classifications (see Table 3A and B), for all cancers combined, and for important subtypes: lymphoid leukaemia, acute nonlymphocytic leukaemia, Hodgkin's disease, and non-Hodgkin lymphoma.

In addition to crude model estimates, adjustment was also made for social/economic indicators by adding a set of minimally correlated indicators into the model. Estimates of rate ratios derived from these models were examined for direction and significance of risk with either increasing distance from the equator (in bands of 10° of latitude) or increasing average annual solar radiation (low, moderate, or high).

## Results

Years of coverage varied (Table 1), for example, Iceland's records started the earliest (1960) and had nearly 30 years of follow-up time, whereas South Korea's registry covered only 3 years and started much later (1992–1994). On average, registries covered 8.51 years (s.d.=3.97), most commonly during the 1980s (see Table 1). Correlations for the economic equality items were all above ∣0.7∣, with the exception of the GINI index which was moderately correlated with the other items (*r* range from ∣0.24∣ to ∣0.34∣). The GINI index and GDP were selected as the covariates for the adjusted model, and both significantly correlated with radiation (GINI *r*=0.64, *P*<0.0001; GDP *r*=−0.43, *P*=0.002) and latitude (GINI *r*=−0.71, *P*<0.0001; GDP *r*=0.53, *P*<0.0001). Because of potential for both over-adjustment and multicollinearity, particularly as these two variables were still moderately correlated (*r*=−0.33, *P*<0.0001), we fit two adjusted models – one with only the GINI index and one with both the GINI index and GDP. Quartiles for GINI index and GDP for each registry are shown in Table 2.

Analyses were performed using negative-binomial models because of a non-zero dispersion parameter. In the initial crude estimates using categorical bands of absolute latitude, all 12 cancer sites as well as the lymphoid leukaemia subtype were significantly (*P*<0.05) associated with latitude (Table 3A). The association with all cancers combined was marginally significant (*P*=0.07) and after adjustment for the GINI index was significant for each cancer type except hepatic tumours, the lymphoid leukaemia and Hodgkins disease subtypes, and all cancers combined. An increased risk was observed with an increase in latitude for leukaemia and both its subtypes, brain and spinal neoplasms, sympathetic nervous system tumours, renal tumours, malignant bone tumours, soft tissue sarcomas, germ cell and gonadal neoplasms, carcinomas and epithelial neoplasms, and all cancer combined. Increasing latitude was associated with a decreased risk for lymphoma and both of its subtypes, retinoblastoma, and other/unspecified neoplasms. After further adjustment for GDP as well as the GINI index, the association between latitude and cancer risk remained significant for only brain and spinal neoplasms, retinoblastoma, renal tumours, soft tissue sarcomas, carcinomas and epithelial tumours, and all cancers combined. Effect sizes were all relatively small, for example, a 10° increase in latitude in the fully adjusted model was associated with an 11% increased risk for brain and spinal neoplasms (RR=1.11; 95% CI 1.07, 1.15). Results were similar for a parallel analysis using the actual latitude rather than a categorical measure (data not shown).

Crude estimates of an increase from moderate to high or low to moderate annual solar radiation were significantly associated with all but three cancer types (hepatic tumours, malignant bone tumours, and carcinomas and epithelial neoplasms; Table 3B). Both leukaemia subtypes were associated with a decreased risk, but neither of the lymphoma subtypes was significantly associated. Risk of all cancers combined was significantly negatively associated with an increase in radiation. After adjustment for the GINI index, the association remained significant for all cancer types except for soft tissue sarcoma, and the association with hepatic tumours remained nonsignificant. Carcinomas and epithelial neoplasms became significantly associated with a decreased risk. Increased solar radiation was associated with a decreased risk for leukaemia and both its subtypes, brain and spinal neoplasms, sympathetic nervous system tumours, renal tumours, malignant bone tumours, germ cell/gonadal neoplasms, and all cancers combined. An increased risk was associated with lymphoma and both its subtypes, retinoblastoma, and other/unspecified neoplasms. After further adjustment for GDP, a decreased risk with increasing solar radiation was still associated with leukaemia and both subtypes, brain and spinal neoplasms, renal tumours, germ cell and gonadal neoplasms, carcinomas and epithelial neoplasms, and all cancers combined (Table 3B). Effect sizes were once again small – an increase in annual solar radiation in the adjusted model was associated with a 14% decrease in risk of germ cell and gonadal neoplasms (RR=0.86; 95% CI 0.79, 0.93). Results were similar for a parallel analysis using actual solar radiation rather than a categorical measure (results not shown).

## Discussion

It has been hypothesised that sun exposure is associated with a decreased risk for cancer due to its associations with the production and absorption of vitamin D (Grant and Mohr, 2009), which is known to have a role in the regulation of genes involved in cell growth and differentiation (Garland *et al*, 2006). There is evidence of an association between sunlight, vitamin D, and adult cancers of various sites (Freedman *et al*, 2002; Boscoe and Schymura, 2006; Lappe *et al*, 2007), and we sought to explore similar associations in childhood cancers. In an unadjusted model, increasing absolute latitude was associated with all cancer types, and annual solar radiation was significantly associated with all but three cancer types. Some of these associations remained significant after adjusting for GDP and GINI indices of registries’ mother nations – most notably in the case of leukaemia, brain and spinal neoplasms, renal tumours, germ cell and gonadal neoplasms, and carcinomas and epithelial neoplasms for solar radiation, and brain and spinal neoplasms, retinoblastoma, renal tumours, soft tissue sarcomas, and carcinomas and epithelial neoplasms with latitude. This indicates that economic disparities among nations could account for some – but not all – of the initial associations; however, it is also possible that there remains some residual confounding or that there are more accurate measures of economic disparities that might account for more of the found association. All cancers combined were also significantly associated with both sunlight surrogates, and in the hypothesised direction, were greater sunlight exposure was associated with a decreased risk. The effect size estimates for the sunlight markers (radiation and latitude) tended to be in opposite directions, which is to be expected as increasing one measure (latitude) is associated with less sunlight, and increasing the other (radiation) is associated with more sunlight, and this consistency between measures strengthens our findings.

The agreement was not perfect, however, and as solar radiation is a direct measure of annual sunlight, this could indicate that latitude is an imperfect substitute measure of sunlight exposure. Indeed, some of the associations between cancer types that were significant for solar radiation were nonsignificant in the models using latitude in the fully adjusted model with both GDP and the GINI index – most notable were the leukaemias and germ cell and gonadal neoplasms. Latitude may reflect more the seasonality of sunlight availability (e.g., Cali, Colombia gets roughly 12 h of sunlight year round, whereas Helsinki, Finland receives roughly 18 h in mid-June, but only about 5.5 h in mid-December (Cornwall, 2010)). Thus, latitude could measure the consistency of daily sunlight throughout the course of the year, whereas solar radiation is a more direct measure of the annual ‘dose’ of sunlight.

Our study has several important limitations. The first is the ‘ecologic fallacy’, inherent in all ecological analyses. Here, even though some geographic areas are subjected to greater or lesser sunlight exposure, we cannot assume that all children within that area are exposed to similar amounts of sunlight. Thus, we cannot measure individual-level sunlight exposure, but instead apply a population-level estimate.

Registries with a wide range of follow-up time could lead to error in terms of consistency of data across this time period, particularly for the socioeconomic indicators. However, most registries provided data for less than a decade's span, primarily in the 1980s, thereby minimising gross error and variability. In addition, our exposure variables latitude and solar radiation have the advantage of being measured with relative accuracy, and are perhaps less prone to error than more complex exposures. The data for solar radiation were not available for our given exposure window, but rather the 22-year period 1983–2005; however, these data were measured with great accuracy and represent an aggregate measure spanning over two decades that overlap our desired timeframe.

As our outcome data come from registries that vary in completeness and accuracy, we adjusted for economic disparities and found that differences in nationwide socioeconomic status between registries did account for some but not all of the estimated effects. It is possible that some residual confounding may still exist. Furthermore, the covariates we utilised to account for economic differences could also be prone to the same inaccuracies as in the cancer registry data, though this was minimised by utilising reliable authorities such as the United Nations for economic factors. There also remains the potential for over-correction, particularly, as our socioeconomic indicator variables were moderately correlated with one another. We have presented results that adjust for only the GINI index as well as results that include both the GINI index and GDP. The fact that the significant associations held for the leukaemias, germ cell tumours, brain and spinal neoplasms, carcinomas and epithelial neoplasms, other/unspecified neoplasms, and all cancers combined even in a fully adjusted model suggests a real association between sunlight and these cancers. As our sunlight proxies were also highly correlated, we presented model results separately, but in our caution to avoid multicollinearity and over-adjustment, some residual confounding is possible. In addition, the rarity of childhood cancers limits the numbers from each registry, unlike studies of adult cancers, which were based on much larger numbers.

Our analyses did not attempt to correct for multiple comparisons, and thus some of our findings may be significant chance alone. We think that this occurring by chance alone is unlikely given the sheer number of estimated effects that were statistically significant. Although many of the estimates were in the hypothesised protective direction (i.e., increased sunlight is associated with a decrease in risk), in particular for the leukaemias, germ cell and gonadal neoplasms, and brain and spinal neoplasms with solar radiation even in the fully adjusted model this was not the case for some cancer types. Most notable were the other/unspecified neoplasms, and lymphoma with solar radiation, and retinoblastoma and other/unspecified neoplasms with latitude, which were significantly associated with increased risk in the fully adjusted models. A lack of consistent findings across tumour types could indicate our findings are artifact, or could simply be indicative of the heterogeneous etiologies of the different cancers. As stated earlier, the opposite direction of estimated effects between the two sunlight surrogates is expected, thus the consistency between our sunlight measures adds strength to our findings despite some of the estimates occurring opposite of the hypothesised direction. The current ecological study provides some support for an association between sunlight exposure and risk of childhood cancer; however, our results should be interpreted with caution as they represent a difficult balance of over and under adjustment of ecological data. An adequately sized case-control study with individual level exposures would be needed to more definitively assess the association between childhood cancer and sunlight.

## Figures and Tables

**Table 1:** Classification of cancer registries by latitude and annual radiation

**Absolute**	**Annual radiation (sum of monthly averages) (kWh m^−2^ per day)**		
**latitude**	**0–45**	**45.1–60**	**60.1–75**
0–10°	—	Nigeria (1985–1992, 70.5, 383), Colombia (1982–1991, 123.7, 514), Costa Rica (1984–1992, 136.3, 1202), Ecuador (1985–1992, 125.5, 368), Singapore-Chinese (1983–1992, 129.3, 587), Singapore-Malay (1968–1992, 89.4, 251), Papua New Guinea (1979–1988, –, 405)	Uganda (1992–1995, 182.7, 340), Brazil-Belem (1987–1991, 101.9, 212)
11–20°	—	Philippines (1983–1992, 100.8, 2727)	Malawi (1991–1995, 182.7, 340), Mali (1991–1995, –, 547), Namibia (1983–1992, 45.9, 241), Zimbabwe (1990–1994, 111.6, 219), Brazil-Goiania (1987–1997, 122.1, 201), Peru (1990–1991, 104.4, 383), Puerto Rico (1983–1991, 118.5, 951), India-Bangalore (1982–1992, 65.2, 881), India-Bombay (1980–1992, 77.3, 2801), India-Madras (1982–1992, 84.2, 1077), India-Poona (1980–1992, 74.0, 664), Thailand (1983–1993, 73.7, 1086)
21–30°	—	Bangladesh (1982–1992, –, 1650), Hong Kong (1980–1989, 132.1, 1639), Vietnam (1991–1994, 109.7, 295)	South Africa-White (1988–1991, –, 875), South Africa-Black (1988–1991, –, 2524), Cuba (1986–1990, 121.8, 1478), United States-Hawaii (1973–1992, 144.2, 193), India-Delhi (1988–1992, 108.1, 1535), Kuwait-Kuwaiti (1983–1989 and 1992–1993, 108.8, 327), Kuwait-non-Kuwaiti (1983–1989 and 1992–1993, 131.1, 325), United Arab Emirates (1984–1992, –, 332)
31–40°	Japan-Osaka (1981–1989,133.4, 2003), New Zealand-Maori (1980–1992, 151.7, 374), New Zealand-non-Maori (1980–1992, 159.4, 1391)	Algeria (1986–1995, 69.8, 370), Uruguay (1988–1992, 120.2, 468), United States-Delaware Valley-White (1980–1989, 144.5, 2018), United States-Delaware Valley-Black (1980–1989, 108.7, 323), United States-SEER-White (1983–1992, 150.3, 5718), United States-SEER-Black (1983–1992, 117.9, 758), China (1981–1992, 104.9, 783), Japan (1980–1992, 116.3, 4476), South Korea (1992–1994, 108.4, 782), Portugal (1989–1992, 159.3, 680), Spain (1980–1991, 141.3, 1885), Spain-Valencia (1983–1990, 145.6, 509), Pakistan (1984–1993, –, 332)	Egypt (1980–1989, 101.4, 1315), United States-Los Angeles-Hispanic (1984–1992, 160.6, 1134), United States-Los Angeles-non-Hispanic White (1984–1992, 163.5, 899), United States-Los Angeles-Black (1984–1992, 113.7, 247), Israel-Jews (1980–1989, 132.8, 1371), Israel-non-Jews (1980–1989, 111.1, 358), Australia (1982–1991, 142.4, 5112)
41–50°	Canada (1982–1991, 149.2, 7260), Croatia (1987–1990, 169.4, 619), Czech Republic (1980–1989, 125.4, 2894), France (1983–1992, 134.8, 2538), France-Lorraine (1983–1992, 138.7, 668), Hungary (1985–1990, 112.1, 1391), Italy-Piedmont (1982–1989, 145.5, 750), Slovakia (1980–1989, 129.2, 1690), Slovenia (1981, 1990, 118.9, 488), Switzerland (1980–1992, 144.4, 931)	United States-New York-White (1983–1991, 148.7, 3584), United States-New York-Black (1983–1991, 109.4, 719), Bulgaria (1980–1989, 99.6, 1906), France-PACA & Corsica (1984–1992, 135.8, 975), Italy (1980–1991, 143.4, 1638)	—
51–60°	Denmark (1983–1991, 158.7, 1241), Estonia (1980–1989, 125.9, 416), Germany-former German Democratic Republic (1981–1989, 127.8, 3653), Germany-former Federal Republic of Germany (1985–1990, 132.0, 7036), Germany (1991–1995, 132.6, 8434), Netherlands (1989–1992, 135.2, 1457), Poland (1980–1989, 113.1, 651), Sweden (1983–1989, 154.3, 1590), the United Kingdom—England and Wales (1981–1990, 122.1, 11352), the United Kingdom-Scotland (1981–1990, 124.9, 1201)	—	—
60–70°	Finland (1980–1989, 153.5, 1420), Iceland (1960–1989, 110.8, 211), Norway (1980–1989, 151.9, 1201)	—	—

Dates of registry, age standardized rate, total number of cases.

**Table 2:** GINI index and gross domestic product quartile for each registry^a^

**GINI**	**GDP**			
**index^b^**	**1**	**2**	**3**	**4**
1	Bangladesh	Cuba, Bulgaria, Czech Republic, Hungary, Poland, Slovakia, Slovenia	Germany-former German Democratic Republic, Germany-former Federal Republic of Germany, Iceland, United Kingdom-England and Wales, United Kingdom-Scotland	Kuwait, Denmark, Finland, Germany, Netherlands, Norway, Sweden, United Arab Emirates
2	China, India-Bangalore, India-Bombay, India-Madras, India-Poona, India-Delhi	Spain	Korea, Italy, Italy-Piedmont, New Zealand	Canada, France, France-Lorraine, France-PACA and Corsica, Japan, Australia
3	Egypt, Vietnam, Pakistan	Algeria, Croatia, Estonia	Israel, Portugal	United States-Greater Delaware Valley, United States-Los Angeles, United States-New York, United States SEER, United States-Hawaii, Japan-Osaka, Switzerland
4	Malawi, Mali, Nigeria, Uganda, Zimbabwe, Colombia, Ecuador, Peru, Philippines, Thailand, Papua New Guinea	Namibia, South Africa, Brazil, Costa Rica, Uruguay, Singapore-Malay	Puerto Rico, Singapore-Chinese, Hong Kong	—

a Calculated for each registry from data from the follow-up period of that registry.

b Lower GINI indicates more equal dispersion of wealth.

**Table 3A:** Estimated rate ratios for association between latitude and childhood cancer

	**Model 1^a^**			**Model 2^b^**			**Model 3^c^**		
**Cancer type**	**Rate ratio^d^**	**95% Confidence interval**	***P*-value**	**Rate ratio^d^**	**95% Confidence interval**	***P*-value**	**Rate ratio^d^**	**95% Confidence interval**	***P*-value**
*Leukaemia*	1.05	1.02, 1.07	^**^	1.07	1.04, 1.11	^**^	1.01	0.97, 1.05	0.58
Lymphoid leukaemia	1.09	1.06, 1.12	^**^	1.12	1.07, 1.17	^**^	1.00	0.95, 1.04	0.87
Acute nonlymphocytic leukaemia	1.02	0.99, 1.05	0.18	1.03	0.99, 1.08	0.13	0.98	0.93, 1.03	0.45
*Lymphoma*	0.86	0.83, 0.89	^**^	0.91	0.86, 0.96	^*^	0.96	0.90, 1.02	0.19
Hodgkins disease	0.99	0.94, 1.03	0.61	0.91	0.86, 0.98	^*^	0.92	0.85, 1.00	0.05
Non-Hodgkin lymphoma	1.02	0.99, 1.06	0.17	0.96	0.91, 1.00	0.07	0.97	0.91, 1.03	0.29
Brain and spinal neoplasms	1.24	1.21, 1.27	^**^	1.24	1.20, 1.28	^**^	1.11	1.07, 1.15	^**^
Sympathetic nervous system tumours	1.18	1.13, 1.23	^**^	1.13	1.06, 1.19	^**^	0.95	0.89, 1.01	0.06
Retinoblastoma	0.80	0.76, 0.84	^**^	0.82	0.76, 0.88	^**^	0.90	0.83, 0.98	0.02
Renal tumours	1.08	1.05, 1.12	^**^	1.18	1.13, 1.24	^**^	1.14	1.08, 1.21	^**^
Hepatic tumours	0.87	0.82, 0.92	^**^	0.94	0.87, 1.02	0.14	0.96	0.87, 1.07	0.45
Malignant bone tumours	1.05	1.02, 1.08	^*^	1.05	1.02, 1.09	^**^	1.01	0.96, 1.06	0.73
Soft tissue sarcoma	0.95	0.91, 0.98	^*^	1.10	1.03, 1.16	^**^	1.10	1.02, 1.17	^**^
Germ cell and gonadal neoplasms	1.06	1.02, 1.10	^*^	1.11	1.05, 1.17	^*^	0.98	0.92, 1.05	0.63
Carcinomas and epithelial neoplasms	0.91	0.86, 0.96	^*^	1.12	1.05, 1.20	^*^	1.09	1.00, 1.19	0.04
Other and unspecified neoplasms	0.77	0.71, 0.83	^**^	0.73	0.66, 0.80	^**^	0.93	0.82, 1.04	0.19
All cancers combined	1.02	1.00, 1.04	0.07	1.07	1.04, 1.10	^**^	1.05	1.02, 1.08	^*^

a Unadjusted estimates.

b Model 1+adjustment for GINI index quartile.

c Model 2+adjustment for gross domestic product quartile.

d For a 10° increase in absolute latitude.

^**^P<0.0001,

^*^P<0.01.

**Table 3B:** Estimated rate ratios for association between annual radiation and childhood cancer

	**Model 1^a^**			**Model 2^b^**			**Model 3^c^**		
**Cancer type**	**Rate ratio^d^**	**95% Confidence interval**	***P*-value**	**Rate ratio^d^**	**95% Confidence interval**	***P*-value**	**Rate ratio^d^**	**95% Confidence interval**	***P*-value**
*Leukaemia*	0.87	0.83, 0.91	^**^	0.88	0.83, 0.92	^**^	0.93	0.88, 0.98	^*^
Lymphoid leukaemia	0.82	0.78, 0.87	^**^	0.84	0.79, 0.89	^**^	0.94	0.88, 1.00	0.04
Acute nonlymphocytic leukaemia	0.90	0.85, 0.95	^*^	0.88	0.82, 0.93	^**^	0.90	0.84, 0.96	^*^
*Lymphoma*	1.24	1.16, 1.33	^**^	1.09	1.02, 1.17	0.02	1.01	0.93, 1.09	0.86
Hodgkins disease	1.08	0.99, 1.17	0.10	1.10	1.00, 1.21	0.04	1.07	0.96, 1.19	0.20
Non-Hodgkin lymphoma	1.02	0.96, 1.09	0.51	1.03	0.97, 1.11	0.33	1.00	0.93, 1.08	0.98
Brain and spinal neoplasms	0.74	0.71, 0.79	^**^	0.78	0.74, 0.82	^**^	0.90	0.85, 0.94	^**^
Sympathetic nervous system tumours	0.85	0.79, 0.92	^**^	0.83	0.77, 0.90	^**^	0.99	0.92, 1.06	0.80
Retinoblastoma	1.38	1.24, 1.54	^**^	1.23	1.10, 1.38	^*^	1.02	0.91, 1.14	0.74
Renal tumours	0.92	0.87, 0.98	0.01	0.87	0.81, 0.93	^**^	0.93	0.86, 0.99	0.04
Hepatic tumours	1.06	0.95, 1.18	0.34	0.94	0.84, 1.06	0.33	0.88	0.77, 1.00	0.05
Malignant bone tumours	0.95	0.91, 1.01	0.08	0.94	0.89, 0.99	0.02	0.99	0.93, 1.05	0.80
Soft tissue sarcoma	1.10	1.03, 1.19	^*^	0.97	0.90, 1.05	0.49	1.00	0.91, 1.09	0.95
Germ cell and gonadal neoplasms	0.82	0.77, 0.89	^**^	0.78	0.73, 0.84	^**^	0.86	0.79, 0.93	^*^
Carcinomas and epithelial neoplasms	0.93	0.84, 1.02	0.13	0.83	0.76, 0.91	^**^	0.86	0.77, 0.96	^*^
Other and unspecified neoplasms	1.67	1.45, 1.93	^**^	1.57	1.36, 1.83	^**^	1.14	0.98, 1.34	0.09
All cancers combined	0.95	0.92, 0.99	^*^	0.92	0.88, 0.96	^**^	0.94	0.90, 0.98	^*^

a Unadjusted estimates.

b Model 1+adjustment for GINI index quartile.

c Model 2+adjustment for gross domestic product quartile.

d For a categorical increase in radiation (category 1: 0–45; category 2: >45–60, category 3: >60 kWh m^−2^ per day).

^**^P<0.0001,

^*^P<0.01.
